# Delay–Energy-Aware Partial Offloading and Coupled Resource Allocation in Hybrid NOMA-MEC Networks: Derivations and Reproducible Evaluation

**DOI:** 10.3390/s26165128

**Published:** 2026-08-13

**Authors:** Jamil K. J. Bataineh, Ahlam Shebli Jawarneh, Khaled F. Hayajneh, Zaid Albataineh

**Affiliations:** 1 Department of Electrical Engineering, Faculty of Engineering, The Hashemite University, Zarqa 13133, Jordan; jamilk@hu.edu.jo; 2 Faculty of Information Technology, Jadara University, Irbid 21110, Jordan; ah.jawarneh@jadara.edu.jo; 3 College of Engineering and Technology, American University of the Middle East, Egaila 54200, Kuwait; khaled.hayajneh@aum.edu.kw; 4 Department of Electronics Engineering, Yarmouk University, Irbid 21163, Jordan

**Keywords:** mobile edge computing, non-orthogonal multiple access, partial offloading, queue-aware scheduling, successive interference cancellation, resource allocation, deep reinforcement learning

## Abstract

This paper considers priority-aware partial computation offloading in an uplink mobile edge computing (MEC) network. Devices assigned to different groups occupy orthogonal subbands, whereas devices within each group use power-domain non-orthogonal multiple access (NOMA) with successive interference cancellation. Task-input size determines the transmitted and processed workload, while queue backlog and application urgency determine the service weight. The Gaussian multiple-access-channel rate region is convex, but the complete allocation problem is not jointly convex in the adopted variables because the offloaded workload is coupled with reciprocal transmission rate and reciprocal edge-CPU allocation. A structure-exploiting block-coordinate projected-gradient method is developed. It combines exact finite-candidate offloading updates, an exact edge-CPU allocation bounded below by deadline feasibility and above by local-path saturation, and an analytical projected power step with Armijo backtracking. For eight users at 23 dBm, pairwise group-based NOMA reduces the weighted delay–energy cost and device energy by 6.18% and 23.44%, respectively, relative to orthogonal access. Queue-aware weighting reduces upper-backlog-quartile delay by 2.69 ms (95% confidence half-width: 0.78 ms) while increasing lower-quartile delay by 8.34 ms (half-width: 2.07 ms). In a paired 15-iteration ablation, generic projected block-coordinate updates have a cost ratio of 1.0098 (half-width: 0.0086) relative to the structured method. A hybrid deep deterministic policy-gradient policy, evaluated over five training seeds, has an 11.77% higher cost while requiring 0.84% of the median online decision time. Of 432 allocations, 392 satisfy the residual-qualified stopping tests and 40 are explicitly reported as iteration-safeguard terminations.

## 1. Introduction

Mobile edge computing (MEC) places computing resources near the radio access network and reduces the dependence of delay-sensitive applications on remote cloud infrastructure [1,2]. During an offloading interval, a device may execute part of a task locally and transmit the remaining input data to an edge server. The service time consequently depends on the task-input size, the supported uplink rate, and the computing resources available at both the device and the edge server.

Power-domain non-orthogonal multiple access (NOMA) allows several users to share a time–frequency resource through superposition and successive interference cancellation (SIC) [3,4,5]. Its theoretical basis lies in the classical multiple-access channel (MAC), including the capacity region, rate splitting, successive decoding, and polymatroid resource allocation [6,7,8,9,10]. In particular, the Gaussian-MAC rate region is convex, and queue-weighted linear rate allocation over that region is a convex optimization problem.

The present study addresses a broader cross-layer problem in which the offloaded workload, transmit powers, and finite edge-CPU shares are selected jointly. The offloaded fraction enters both the transmission delay and the edge-processing delay. These workload–rate and workload–CPU couplings are not jointly convex in the original variables, even when the rate variables are constrained to the convex MAC region. A fixed SIC order also couples individual rates through the powers of users decoded later. The radio-capacity structure and the curvature of the complete computation-offloading problem must therefore be treated separately.

Resource allocation must also reflect differences in user demand. Maximum-weight scheduling establishes queue backlog as a principled basis for service priority [11]. In the proposed model, task size determines the number of bits transmitted and processed, whereas queue backlog and application urgency determine the service weight. Channel gain remains a separate quantity used for grouping and SIC ordering. The allocation is determined at the beginning of the interval, and the task-completion metric contains only the local and remote service paths.

NOMA–MEC research has considered time and power allocation, partial offloading, energy reduction, fairness, hybrid access, and learning-based control [12,13,14,15,16,17,18,19]. Recent studies include wireless-powered MEC, relay- and cache-assisted NOMA–MEC, secure learning-based allocation, and joint time–energy optimization [20,21,22,23,24]. Within this literature, priority-aware group-based partial offloading requires three elements to be considered together: a direct connection between task demand and radio support, a mathematical distinction between convex MAC scheduling and cross-layer resource coupling, and a solution method that uses the available subproblem structure with residual-qualified termination.

The contributions of this paper are as follows.

1. A priority-aware group-based NOMA–MEC model links the task-input size to the transmitted and computed workload and uses queue backlog and application urgency to define service weights. OMA, pairwise group-based NOMA (H-NOMA), and single-group power-domain NOMA (P-NOMA) are represented by the same grouping model.
2. The Gaussian-MAC rate region is shown to be convex, while the joint partial-offloading formulation is shown to be non-convex in the adopted workload, rate, and edge-CPU variables. The result identifies the cross-layer delay terms, rather than the multiple-access region, as the source of non-convexity.
3. A structure-exploiting block-coordinate projected-gradient method combines exact cyclic offloading-coordinate updates, an exact deadline-bounded square-root edge-CPU allocation, and an analytical projected power update. The edge-CPU solution includes both the deadline-induced lower bound and the local-path upper cap. Residual-qualified tests determine convergence; runs that reach the iteration limit are identified separately.
4. Numerical experiments compare the three access structures, queue-aware and uniform weighting, three SIC orders on a common feasible set, a generic projected block-coordinate method, equal allocation, and a five-seed hybrid DDPG policy. Energy-weight and edge-capacity sensitivity and user-count scalability are also quantified.

The remainder of the paper is organized as follows. Section 2 reviews the foundations and recent NOMA–MEC literature. Section 3 defines the service, communication, and computation models. Section 4 formulates the optimization problem and establishes its curvature. Section 5 derives SBCD-PG, and Section 6 defines the learning-based benchmark. Section 7 presents the numerical study. Section 8 concludes the paper.

## 2. Related Work and Positioning

### 2.1. Gaussian MAC, SIC, and Queue-Based Priority

The foundations of multiuser access include the capacity formulations of Ahlswede and Liao [6,7], rate splitting and successive decoding for the Gaussian MAC [8], and the polymatroid resource-allocation structure characterized by Tse and Hanly [9]. Iterative water filling subsequently provided a distributed optimization framework for Gaussian vector MACs [10]. These results establish the convexity of the achievable MAC region and provide the basis for weighted rate allocation.

Power-domain NOMA research has focused on user multiplexing, power control, pairing, and SIC order [3,4,5]. The decoding order determines the residual interference present at each SIC stage. Queue-based priority addresses a different aspect of the allocation: maximum-weight scheduling uses queue state to favor users with greater service demand [11]. The present model therefore places backlog and urgency in the objective weights and examines SIC ordering separately.

### 2.2. Optimization-Based MEC and NOMA–MEC

Joint communication and computation allocation in MEC is generally coupled because radio resources, task partitioning, and edge-CPU resources interact [1,2,25]. In NOMA-assisted MEC, Wu et al. optimized offloading and time allocation [12], and Ding et al. studied joint power and time allocation [13]. Yang et al. minimized a weighted completion-time and energy objective, with a convex reformulation available for a special infinite-cloud-capacity case [14]. Fang et al. treated partial offloading and transformed a non-convex delay problem into an equivalent quasi-convex form [15]. These studies show that the curvature depends on the selected variables and resource assumptions: radio-only and special MEC formulations may be convex or quasi-convex, whereas joint finite-resource formulations may remain non-convex.

Later work incorporated changing system states, subchannel assignment, hybrid access, and fairness. Tuong et al. used deep reinforcement learning for partial offloading [16], and Wang et al. applied DDPG to joint resource management for latency minimization [26]. Liu et al. jointly optimized energy, subchannels, power, and computing resources under latency constraints [17]. Ding et al. developed a hybrid NOMA offloading framework [18], and Kumar et al. proposed a convergent max–min task-offloading method [19]. These studies motivate the group-based access model and the use of problem-specific subproblem updates.

### 2.3. Recent and Learning-Based Resource Allocation

Recent studies continue to broaden the NOMA–MEC design space. Chen et al. considered computation-time minimization in wireless-powered NOMA–MEC [20]. At IEEE GLOBECOM 2024 in Cape Town, Wang et al. studied relay-assisted NOMA–MEC resource allocation [21]; the associated workshops included an energy-efficient cache-assisted NOMA–MEC allocation method [22]. Zhang et al. developed a multi-DNN architecture for secure MEC offloading and resource allocation [23], while Liu et al. addressed partial offloading with joint time and energy optimization [24].

Deep reinforcement learning is useful when resource decisions must be made repeatedly under changing conditions. Deterministic policy gradients are particularly suitable for continuous power and resource variables [27]. In the per-interval setting considered here, the current allocation does not affect the next channel or task state. The learning benchmark is therefore formulated as a one-step terminal decision that maps the observed state directly to an allocation.

Table 1 summarizes the relationship between the representative literature and the present study.

## 3. System and Service Model

### 3.1. Task Model and Service Priority

Consider one access point equipped with an MEC server and a set K={1, …, K} of single-antenna devices. At the beginning of an allocation interval, device *i* presents one admitted task described by

(1) $$T_{i}=\left(L_{i},\;c_{i},\;D_{i},\;Q_{i},\;u_{i}\right),\;$$

where $L_{i}$ is the input size, $c_{i}$ is the required number of CPU cycles per input bit, $D_{i}$ is the completion deadline, $Q_{i}$ is the queued workload at the beginning of the interval, and $u_{i}\geq 1$ is an application-urgency factor. The input may represent, for example, a sensor-data block, an image segment, or a control-data batch. The output returned by the edge server is assumed to be much smaller than the input, and its downlink delay is neglected.

The partial-offloading ratio $\rho_{i}\in \left[0,\;1\right]$ divides the task into $\rho_{i}L_{i}$ bits for remote execution and $\left(1-\rho_{i}\right)L_{i}$ bits for local execution. The service weight is

(2) $${\tilde{w}}_{i}=u_{i}\left(1+\eta_{Q}\frac{Q_{i}}{Q_{0}}\right),\;\;w_{i}=\frac{{\tilde{w}}_{i}}{\sum_{j\in K}{\tilde{w}}_{j}},\;$$

where $Q_{0}$ is a backlog normalization constant and $\eta_{Q}\geq 0$ controls the influence of queue state. Thus, $L_{i}$ determines the transmitted and computed workload, while $\left(Q_{i},\;u_{i}\right)$ determine service priority. Uniform weighting is recovered by setting $w_{i}=1/K$.

The resource decision is determined at the beginning of the interval. The task-completion time defined below comprises local processing and the remote path formed by uplink transmission and edge execution. The time required to determine the allocation is not added to these service components.

**Figure 1:**
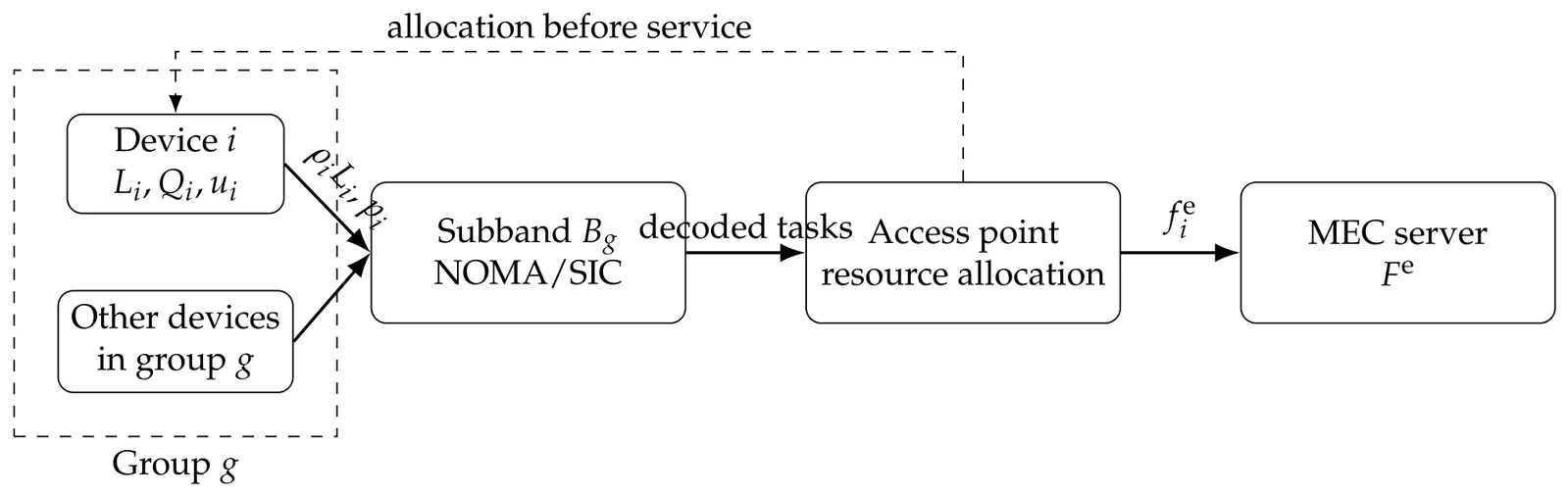
Service workflow within one allocation interval. Task-input bits determine the offloaded payload, while queue backlog and urgency determine the service weight.

Figure 1 summarizes the service workflow and the resource decisions made before task execution.

### 3.2. Group-Based Uplink Multiple Access

The devices are partitioned into *G* disjoint groups ${\left\{K_{g}\right\}}_{g=1}^{G}$. Each group occupies an orthogonal subband of width

(3) $$B_{g}=\frac{B}{G}.$$

Users in the same group transmit simultaneously using power-domain NOMA. This definition produces three access structures:

- OMA: G=K and every group is a singleton;
- H-NOMA: the strongest and weakest remaining channels are paired, giving groups of size at most two;
- P-NOMA: G=1 and all devices share one subband.

The H-NOMA structure limits the number of signals processed in each SIC chain while retaining simultaneous transmission within each pair. Group membership is fixed during one allocation interval.

Let $h_{i}$ be the complex uplink coefficient and $g_{i}={\left|h_{i}\right|}^{2}$ its power gain. The transmit power is $p_{i}$, and the noise in one group is $n_{g}=N_{0}B_{g}F_{\mathrm{NF}}$, where $N_{0}$ is the thermal-noise density and $F_{\mathrm{NF}}$ is the receiver noise factor. For group *g*, let

(4) $$\pi_{g}\left(1\right),\;\pi_{g}\left(2\right),\;\ldots ,\;\pi_{g}\left(\right|K_{g}\left|\right)$$

be the SIC decoding order. The signal at position *r* is decoded while signals at positions $r+1,\;\ldots ,\;|K_{g}|$ remain as interference. For $i=\pi_{g}\left(r\right)$,

(5) $$\begin{array}{cc} S_{i}\left(p\right) & =n_{g}+\sum_{l=r}^{|K_{g}|}p_{\pi_{g}\left(l\right)}g_{\pi_{g}\left(l\right)},\; \end{array}$$

(6) $$\begin{array}{cc} I_{i}\left(p\right) & =n_{g}+\sum_{l=r+1}^{|K_{g}|}p_{\pi_{g}\left(l\right)}g_{\pi_{g}\left(l\right)},\; \end{array}$$

(7) $$\begin{array}{cc} R_{i}\left(p\right) & =B_{g}\log_{2}\;\left(\frac{S_{i}\left(p\right)}{I_{i}\left(p\right)}\right). \end{array}$$

The default order sorts users by decreasing $g_{i}$ within each group, so a strong signal is decoded and removed before weaker signals are detected. The order determines the residual-interference set in (7). Section 7.4 compares this channel-gain order with priority-based and reverse-priority alternatives.

### 3.3. Local, Radio, and Edge Processing

Device *i* has a fixed local CPU frequency $f_{i}^{\mathrm{loc}}$. Its local execution time is

(8) $$T_{i}^{\mathrm{loc}}\left(\rho_{i}\right)=\frac{\left(1-\rho_{i}\right)c_{i}L_{i}}{f_{i}^{\mathrm{loc}}}.$$

The uplink and edge execution times are

(9) $$\begin{array}{cc} T_{i}^{\mathrm{tx}}\left(\rho_{i},\;p\right) & =\frac{\rho_{i}L_{i}}{R_{i}\left(p\right)},\; \end{array}$$

(10) $$\begin{array}{cc} T_{i}^{e}\left(\rho_{i},\;f_{i}^{e}\right) & =\frac{\rho_{i}c_{i}L_{i}}{f_{i}^{e}},\; \end{array}$$

where $f_{i}^{e}\geq 0$ is the edge-CPU allocation and $\sum_{i}f_{i}^{e}\leq F^{e}$. Local and remote task fractions execute in parallel; therefore,

(11) $$T_{i}=\max\left\{T_{i}^{\mathrm{loc}},\;\;T_{i}^{\mathrm{tx}}+T_{i}^{e}\right\}.$$

The standard dynamic-voltage local energy model and the uplink transmit energy are

(12) $$\begin{array}{cc} E_{i}^{\mathrm{loc}}\left(\rho_{i}\right) & =\kappa_{i}{\left(f_{i}^{\mathrm{loc}}\right)}^{2}\left(1-\rho_{i}\right)c_{i}L_{i},\; \end{array}$$

(13) $$\begin{array}{cc} E_{i}^{\mathrm{tx}}\left(\rho_{i},\;p\right) & =p_{i}T_{i}^{\mathrm{tx}}=\frac{p_{i}\rho_{i}L_{i}}{R_{i}\left(p\right)}. \end{array}$$

Circuit, downlink-result, and MEC-server electricity are omitted to keep the per-device decision model identifiable. Their inclusion is a direct extension but changes the interpretation of the energy objective.

## 4. Problem Formulation and Curvature

Introduce an epigraph variable $z_{i}$ for completion time and a positive conversion factor $\lambda_{E}$ in seconds per joule. The weighted delay–energy problem is

(14) $$\begin{array}{cc} \underset{p,\;\rho ,\;f^{e},\;z}{\mathrm{minimize}}\; & J=\sum_{i\in K}w_{i}\left[z_{i}+\lambda_{E}\left(E_{i}^{\mathrm{loc}}+E_{i}^{\mathrm{tx}}\right)\right] \end{array}$$

(15) $$\begin{array}{cccc} \mathrm{subject}\;\mathrm{to}\; & z_{i}\geq T_{i}^{\mathrm{loc}}\left(\rho_{i}\right),\; & \; & i\in K,\; \end{array}$$

(16) $$\begin{array}{cccc} \; & z_{i}\geq T_{i}^{\mathrm{tx}}\left(\rho_{i},\;p\right)+T_{i}^{e}\left(\rho_{i},\;f_{i}^{e}\right),\; & \; & i\in K,\; \end{array}$$

(17) $$\begin{array}{cccc} \; & z_{i}\leq D_{i},\; & \; & i\in K,\; \end{array}$$

(18) $$\begin{array}{cccc} \; & 0\leq \rho_{i}\leq 1,\;\;0\leq p_{i}\leq P_{i}^{\max},\; & \; & i\in K,\; \end{array}$$

(19) $$\begin{array}{cc} \; & f_{i}^{e}\geq 0,\;\;\sum_{i\in K}f_{i}^{e}\leq F^{e}. \end{array}$$

When $\rho_{i}=0$, the transmission and edge-processing times are defined as zero and the corresponding edge-CPU allocation may be zero. A positive offloaded fraction requires a positive transmission rate and a positive edge-CPU allocation.

The objective measures priority-weighted service delay and device energy. The factor $\lambda_{E}$ converts energy into a time-equivalent cost, so *J* is measured in seconds. The deadline constraints connect the workload to the radio and computing resources: a larger task or a shorter deadline narrows the feasible offloading interval, while a larger backlog or urgency factor increases the task’s weight in the objective.

### 4.1. Convex Gaussian-MAC Region

For a group $K_{g}$, introduce rate variables $r_{g}$. The Gaussian-MAC rate–power region is

(20) $$C_{g}=\left\{\left(r_{g},\;p_{g}\right):\sum_{i\in S}r_{i}\leq B_{g}\log_{2}\;\left(1+\frac{\sum_{i\in S}g_{i}p_{i}}{n_{g}}\right),\;\;\forall \;S\subseteq K_{g}\right\}.$$

For every subset S, the left side minus the logarithmic term is convex in $\left(r_{g},\;p_{g}\right)$. Hence, $C_{g}$ is convex and has the polymatroid structure described in [9,10]. Maximizing a queue-weighted linear rate utility over this region is therefore a convex optimization problem. With a fixed offloaded workload, $L_{i}/r_{i}$ is also convex for $r_{i}>0$.

### 4.2. Curvature of the Joint Offloading Problem

The complete problem optimizes the workload fraction jointly with radio and CPU resources. The following result characterizes the resulting curvature in the adopted variables.

**Proposition 1.** 
*Even if the rates are represented by variables constrained to the convex Gaussian-MAC region, Problem *(14) *is not jointly convex in the adopted variables $\left(\rho ,\;r,\;f^{e}\right)$.*

**Proof.**  For one user, define

(21) $$\phi_{i}\left(\rho_{i},\;r_{i}\right)=\frac{\rho_{i}L_{i}}{r_{i}},\;\;r_{i}>0.$$

Its Hessian is

(22) $$\nabla^{2}\phi_{i}=L_{i}\left[\begin{array}{cc} 0 & -r_{i}^{-2}\\ -r_{i}^{-2} & 2\rho_{i}r_{i}^{-3} \end{array}\right],\;\;\det\left(\nabla^{2}\phi_{i}\right)=-\frac{L_{i}^{2}}{r_{i}^{4}}<0.$$

The Hessian is indefinite, so the transmission-delay term is neither jointly convex nor jointly concave in $\left(\rho_{i},\;r_{i}\right)$. Independently, with $C_{i}=c_{i}L_{i}$,

(23) $$\psi_{i}\left(\rho_{i},\;f_{i}^{e}\right)=\frac{C_{i}\rho_{i}}{f_{i}^{e}}$$

has

(24) $$\det\left(\nabla^{2}\psi_{i}\right)=-\frac{C_{i}^{2}}{{\left(f_{i}^{e}\right)}^{4}}<0.$$

Thus, the edge-delay term is also not jointly convex. These terms occur in the remote-path epigraph constraint, whose defining function is consequently non-convex in the stated variables. The convexity of the MAC region therefore does not make the full joint workload–rate–CPU formulation convex. □

The maximum in (11) is represented by the epigraph constraints (15) and (16); the non-convexity arises from the remote-path function in (16). Convex and quasi-convex transformations remain available for several special MEC formulations [14,15].

### 4.3. Fixed-SIC Rate Jacobian

The fixed-SIC corner used in (7) couples user delays through transmit power. For user $i=\pi_{g}\left(r\right)$ and any $k\in K_{g}$,

(25) $$\frac{\partial R_{i}}{\partial p_{k}}=\frac{B_{g}g_{k}}{\ln2}\left(\frac{1\{{\mathrm{pos}}_{g}\left(k\right)\geq r\}}{S_{i}}-\frac{1\{{\mathrm{pos}}_{g}\left(k\right)>r\}}{I_{i}}\right),\;$$

where ${\mathrm{pos}}_{g}\left(k\right)$ denotes the position of user *k* in the decoding order and 1{·} is the indicator function.

Using (9),

(26) $$\frac{\partial T_{i}^{\mathrm{tx}}}{\partial p_{k}}=-\frac{\rho_{i}L_{i}}{R_{i}^{2}}\frac{\partial R_{i}}{\partial p_{k}}.$$

For two users, the Hessian of the first decoded user’s individual rate has a negative determinant; Appendix A provides the expression. Thus, an individual fixed-order SIC rate is not generally jointly concave in all transmit powers, although the full MAC rate region is convex.

## 5. Structure-Exploiting Block-Coordinate Method

The method alternates among the offloading-ratio, edge-CPU, and transmit-power blocks. For fixed powers and CPU allocations, each offloading coordinate has a piecewise-affine objective and is minimized over the feasible endpoints and the point at which the local and remote paths are balanced. For fixed powers and offloading ratios, the edge-CPU block has a bounded square-root solution obtained from its KKT conditions; its lower bound enforces the remote-path deadline, and its upper cap marks the point beyond which the local path dominates. The transmit powers remain mutually coupled through the SIC rates and are updated by a projected-gradient step using the complete analytical rate Jacobian. Each trial power vector is followed by two ratio/CPU sweeps and is accepted only when it is feasible and satisfies the Armijo condition. The resulting procedure is denoted by SBCD-PG.

### 5.1. Exact Cyclic Offloading-Coordinate Updates

For fixed $\left(p,\;f^{e}\right)$, define

(27) $$\begin{array}{cc} a_{i} & =\frac{c_{i}L_{i}}{f_{i}^{\mathrm{loc}}},\; \end{array}$$

(28) $$\begin{array}{cc} b_{i} & =\frac{L_{i}}{R_{i}\left(p\right)}+\frac{c_{i}L_{i}}{f_{i}^{e}},\; \end{array}$$

(29) $$\begin{array}{cc} e_{i}^{0} & =\kappa_{i}{\left(f_{i}^{\mathrm{loc}}\right)}^{2}c_{i}L_{i},\; \end{array}$$

(30) $$\begin{array}{cc} e_{i}^{1} & =\frac{p_{i}L_{i}}{R_{i}\left(p\right)}. \end{array}$$

The user contribution is

(31) $$q_{i}\left(\rho_{i}\right)=w_{i}\left[\max\left\{a_{i}\left(1-\rho_{i}\right),\;b_{i}\rho_{i}\right\}+\lambda_{E}\left\{e_{i}^{0}\left(1-\rho_{i}\right)+e_{i}^{1}\rho_{i}\right\}\right].$$

Deadline feasibility gives

(32) $${\underline{\rho }}_{i}=\max\left\{0,\;1-\frac{D_{i}}{a_{i}}\right\},\;\;{\bar{\rho }}_{i}=\min\left\{1,\;\frac{D_{i}}{b_{i}}\right\}.$$

Because (31) is piecewise affine, its exact coordinate minimizer belongs to

(33) $$R_{i}=\left\{{\underline{\rho }}_{i},\;\;{\bar{\rho }}_{i},\;\;{\mathrm{proj}}_{\left[{\underline{\rho }}_{i},\;{\bar{\rho }}_{i}\right]}\left(\frac{a_{i}}{a_{i}+b_{i}}\right)\right\}.$$

Accordingly,

(34) $$\rho_{i}^{+}=\underset{r\in R_{i}}{arg min}q_{i}\left(r\right).$$

The candidate comparison accounts for both the local–remote path balance and the energy term.

### 5.2. Exact Edge-CPU Block

For fixed (p, ρ), let

(35) $$d_{i}=\rho_{i}c_{i}L_{i},\;\;l_{i}=T_{i}^{\mathrm{loc}},\;\;t_{i}=T_{i}^{\mathrm{tx}}.$$

For an active offloaded workload $d_{i}>0$, remote-path deadline feasibility requires $t_{i}<D_{i}$ and

(36) $$f_{i}^{\min}=\frac{d_{i}}{D_{i}-t_{i}}.$$

If $l_{i}>t_{i}$, allocating more than

(37) $${\bar{f}}_{i}=\frac{d_{i}}{l_{i}-t_{i}}$$

produces no latency benefit because the local path becomes dominant. If $l_{i}\leq t_{i}$, set ${\bar{f}}_{i}=+\infty$. The KKT solution of the active remote-delay subproblem is

(38) $$f_{i}^{e}\left(\nu \right)=\min\left\{{\bar{f}}_{i},\;\;\max\left[f_{i}^{\min},\;\sqrt{\frac{w_{i}d_{i}}{\nu }}\right]\right\},\;$$

where ν>0 is selected so that $\sum_{i}f_{i}^{e}\left(\nu \right)=F^{e}$ whenever the budget is active. The monotonicity of the allocation in ν permits a scalar bisection. If every active upper cap is finite and their sum is below the budget, allocation at the caps is optimal and the remaining CPU capacity is unused.

The fixed-(p, ρ) block is feasible only if

(39) $$l_{i}\leq D_{i}\;\forall i,\;\;t_{i}<D_{i}\;\forall i:d_{i}>0,\;\;\sum_{i:d_{i}>0}f_{i}^{\min}\leq F^{e}.$$

These conditions also imply $f_{i}^{\min}\leq {\bar{f}}_{i}$ whenever ${\bar{f}}_{i}$ is finite. An inactive user has $f_{i}^{e}=0$. If (39) fails, the associated ratio or trial-power point is infeasible and is not accepted by the outer procedure. Thus, (38) is the exact feasible solution of the full fixed-ratio CPU subproblem, rather than a solution of its budget constraint alone.

### 5.3. Coupled Projected Power Block

Let

(40) $$\chi_{i}=\left\{\begin{array}{cc} 1,\; & T_{i}^{\mathrm{tx}}+T_{i}^{e}>T_{i}^{\mathrm{loc}},\;\\ 0,\; & T_{i}^{\mathrm{tx}}+T_{i}^{e}<T_{i}^{\mathrm{loc}},\;\\ 1/2,\; & T_{i}^{\mathrm{tx}}+T_{i}^{e}=T_{i}^{\mathrm{loc}},\; \end{array}\right.$$

be a valid selection from the subgradient of the maximum. The power derivative of *J* is

(41) $$\frac{\partial J}{\partial p_{k}}=\sum_{i\in K}w_{i}\left[\chi_{i}\frac{\partial T_{i}^{\mathrm{tx}}}{\partial p_{k}}+\lambda_{E}\left(1\left\{i=k\right\}T_{i}^{\mathrm{tx}}+p_{i}\frac{\partial T_{i}^{\mathrm{tx}}}{\partial p_{k}}\right)\right].$$

Powers are normalized as $x_{i}=p_{i}/P_{i}^{\max}$. The projected update is evaluated on $x_{i}\in \left[x_{\min},\;1\right]$, where the small positive floor avoids the singular zero-rate boundary for an active offloaded task; $x_{\min}=10^{-3}$ in the numerical study. Let

(42) $$g_{x}^{t}=\mathrm{diag}\left(P^{\max}\right)\nabla_{p}J,\;\;d^{t}=-\frac{g_{x}^{t}}{\max\{∥g_{x}^{t}{∥}_{\infty },\;\delta_{g}\}},\;$$

where $\delta_{g}>0$ prevents division by zero. A trial point is

(43) $$x\left(\alpha \right)={\mathrm{proj}}_{{\left[x_{\min},\;1\right]}^{K}}\left(x^{t}+\alpha d^{t}\right).$$

After each trial power step, the ratio and CPU blocks are updated before the objective is evaluated. The step is accepted if it is feasible and satisfies

(44) $$J\left(x\left(\alpha \right)\right)\leq J\left(x^{t}\right)+c_{A}\nabla_{x}J{\left(x^{t}\right)}^{T}\left(x\left(\alpha \right)-x^{t}\right),\;$$

with $c_{A}\in \left(0,\;1\right)$. Otherwise, α is reduced geometrically.

### 5.4. Termination and Computational Complexity

Termination is governed by the objective change, the allocation change, and the projected-gradient mapping. Define

(45) $$\begin{array}{cc} r_{J}^{t} & =\frac{|J^{t}-J^{t-1}|}{\max\{|J^{t-1}|,\;10^{-12}\}},\; \end{array}$$

(46) $$\begin{array}{cc} r_{x}^{t} & =\max\left\{∥x^{t}-x^{t-1}{∥}_{\infty },\;{∥\rho^{t}-\rho^{t-1}∥}_{\infty },\;\frac{∥f^{t}-f^{t-1}{∥}_{\infty }}{F^{e}}\right\},\; \end{array}$$

(47) $$\begin{array}{cc} r_{\mathrm{PG}}^{t} & ={∥x^{t}-{\mathrm{proj}}_{{\left[x_{\min},\;1\right]}^{K}}\left(x^{t}-\nabla_{x}J^{t}\right)∥}_{\infty }. \end{array}$$

The strict residual test requires $r_{J}^{t}\leq \epsilon_{J}$, $r_{x}^{t}\leq \epsilon_{x}$, and $r_{\mathrm{PG}}^{t}\leq \epsilon_{\mathrm{PG}}$ in two consecutive iterations. A second test combines the relative improvement over a window of *W* iterations with the same projected-gradient requirement:

(48) $$\frac{J^{t-W}-J^{t}}{\max\{|J^{t-W}|,\;10^{-12}\}}\leq \epsilon_{W}\;\mathrm{and}\;r_{\mathrm{PG}}^{t}\leq \epsilon_{\mathrm{PG}}.$$

Here, $r_{\mathrm{PG}}^{t}$ is the box-projected diagnostic for the coupled power block, not a complete KKT residual for every constraint of the joint problem. The algorithm declares residual-qualified termination only when one of these two tests is satisfied. The value $I_{\max}$ is checked last as a finite safeguard; an allocation returned at that limit is reported as a safeguard termination and is not described as a stationary point.

Algorithm 1 summarizes the complete SBCD-PG allocation procedure and its termination logic.
**Algorithm 1** Structured block-coordinate projected-gradient allocation.**Input:** task and channel state; access structure; tolerances $\epsilon_{J},\;\epsilon_{x},\;\epsilon_{\mathrm{PG}},\;\epsilon_{W}$; window *W*; safeguard $I_{\max}$. Form the groups and SIC order, and initialize feasible $p^{0},\;\rho^{0},\;f^{0}$. Apply the ratio update, CPU update, and a second ratio/CPU sweep; evaluate $J^{0}$. For $t=1,\;2,\;\ldots ,\;I_{\max}$:  (a) Save $\left(p^{t-1},\;\rho^{t-1},\;f^{t-1},\;J^{t-1}\right)$.  (b) Update each $\rho_{i}$ by minimizing (31) over (33).  (c) Check (39); update $f^{e}$ using (38), followed by a second ratio/CPU sweep.  (d) Evaluate (25) and (41).  (e) Backtrack the projected power step until feasibility and (44) hold.  (f) Recompute the ratio and CPU blocks and evaluate $J^{t}$.  (g) Stop with residual-qualified status if the strict test holds twice or (48) holds; otherwise continue to the safeguard. Return $\left(p,\;\rho ,\;f^{e}\right)$ and the termination residuals.


Each feasible ratio coordinate and CPU block is non-increasing in the objective for fixed values of the remaining variables, and the Armijo rule accepts only a non-increasing feasible power step. Since J≥0, the accepted objective sequence converges. On the evaluated positive-rate domain $x_{i}\in \left[x_{\min},\;1\right]$, if the iterations are continued and the standard continuity, compactness, and regularity conditions for block successive minimization hold, accumulation points for which all block residuals vanish are stationary [28,29]. This asymptotic statement does not convert an iteration-safeguard return into a stationarity certificate, and the method does not guarantee a global optimum.

For a maximum group size $M_{g}$, rate/Jacobian evaluation requires $O(KM_{g})$ arithmetic with suffix sums and at most $O(K^{2})$ for a single P-NOMA group. The ratio block is O(K), and the CPU block is $O(K\log\left(1/\epsilon_{\nu }\right))$. If $N_{\mathrm{LS}}$ trial steps are tested, one outer iteration costs approximately

(49) $$O\left(N_{\mathrm{LS}}KM_{g}+N_{\mathrm{LS}}K\log\left(1/\epsilon_{\nu }\right)\right),\;$$

with $M_{g}\leq 2$ for the H-NOMA configuration.

## 6. Hybrid DDPG Allocation Benchmark

Deep reinforcement learning provides a direct state-to-allocation map after training and is therefore relevant when similar resource-allocation decisions are repeated. The present model is defined for one allocation interval: the selected action does not influence a subsequent channel, battery, or queue state. Each interval is consequently represented as a one-step episode with a terminal reward. Because every transition is terminal, the critic target is the observed reward without a bootstrapped next-state term, and the actor maximizes the learned critic. The learned component is therefore an amortized one-step allocator rather than a model of temporal control.

For *K* devices, the normalized state has dimension 5K and contains the channel gains, task sizes, queue backlogs, local CPU frequencies, and service weights. The 3K continuous outputs of the actor represent transmit-power fractions, offloading ratios, and edge-CPU weights. The actor contains two hidden layers with 128 neurons each, and the critic contains hidden layers with 192 and 128 neurons. Following the deterministic policy-gradient framework [27], the terminal reward is

(50) $$r=-\frac{J}{J_{0}}-\zeta {\left(\max_{i}\frac{{\left[T_{i}-D_{i}\right]}_{+}}{D_{i}}\right)}^{2},\;$$

where $J_{0}>0$ is a normalization constant and ζ>0 penalizes deadline violations.

The actor proposes the powers and initial offloading and CPU variables, which are first mapped to their resource bounds. Two analytical offloading-ratio and edge-CPU sweeps from Section 5.1 and Section 5.2 then select deadline-compatible ratios, refine the CPU allocation, and enforce its budget; feasibility is checked after the second sweep. The projected power-gradient block is not used. The benchmark is therefore a hybrid learned allocation policy, rather than an end-to-end unconstrained DDPG output. It is compared with SBCD-PG and equal allocation in terms of weighted cost, feasibility, seed variability, and relative online decision time.

## 7. Numerical Evaluation

### 7.1. Numerical Setup

The urban macrocell channel has path loss $128.1+37.6\log_{10}\left(d_{\mathrm{km}}\right)$ dB and independent Rayleigh fading. Unless a specific sweep is stated, K=8, B=10 MHz, $F^{e}=40$ GHz, $c_{i}=500$ cycles/bit, and $P_{i}^{\max}=23$ dBm. Table 2 lists the remaining settings. The H-NOMA groups pair the weakest and strongest remaining users, and the default SIC order is decreasing channel gain.

The experiments use independent channel and task states and report normal-approximation 95% confidence intervals. The power sweep applies all six power limits and three access structures to the same 24 states, giving 3×6×24=432 paired allocations. The priority, SIC-order, and allocation-method comparisons each use 60 states. The hybrid DDPG policy is trained independently from five seeds for 6000 one-step episodes per seed and evaluated on 60 states not used during training. The reported learned-policy result averages the five policies on the same test states. Relative decision times are normalized to SBCD-PG within the same evaluation.

### 7.2. Access-Mode Delay–Energy Performance

**Figure 2:**
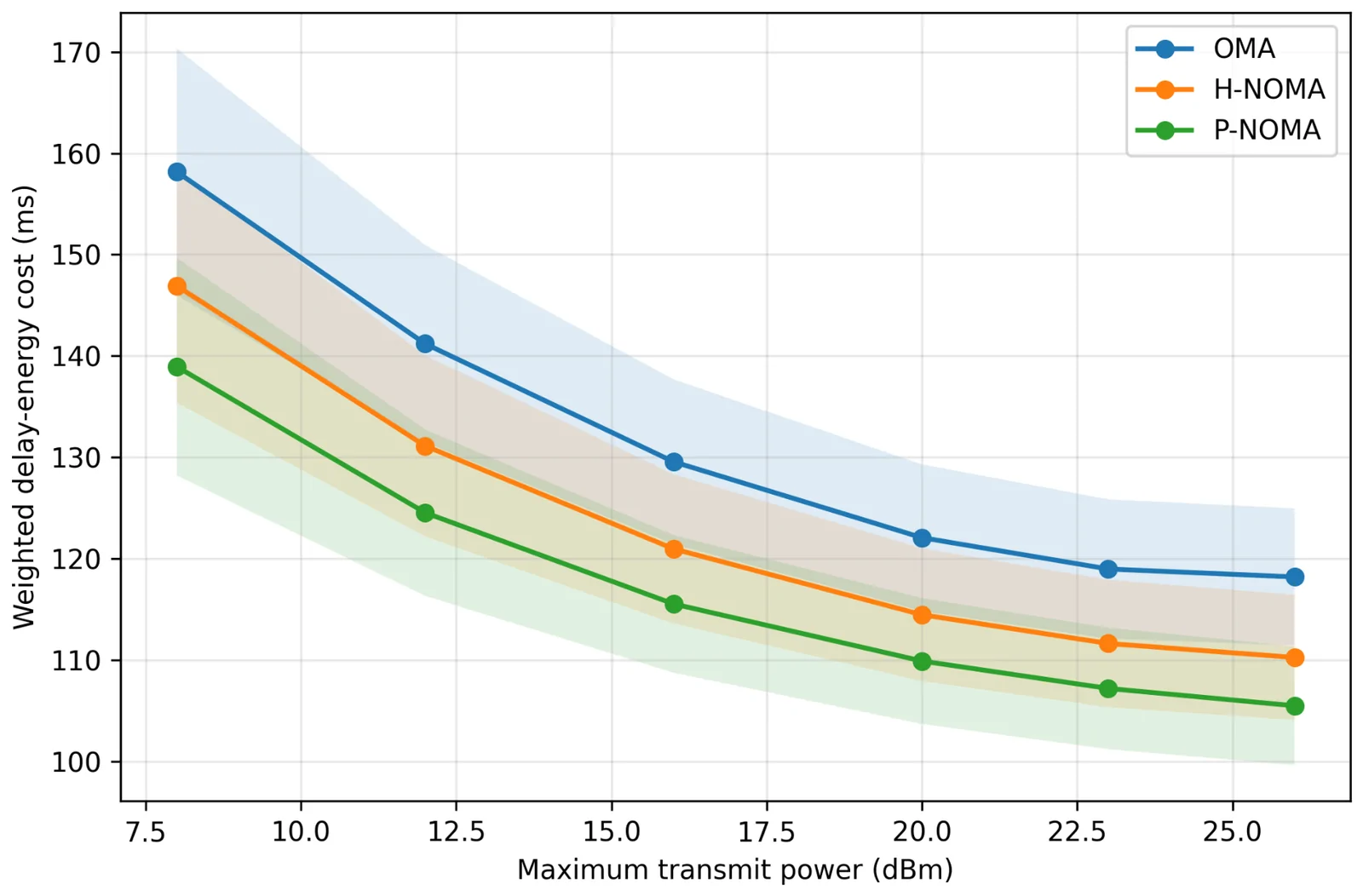
Priority-weighted delay–energy cost versus maximum transmit power. Solid lines with circular markers show sample means, and shaded bands show 95% confidence intervals over 24 states per point.

**Figure 3:**
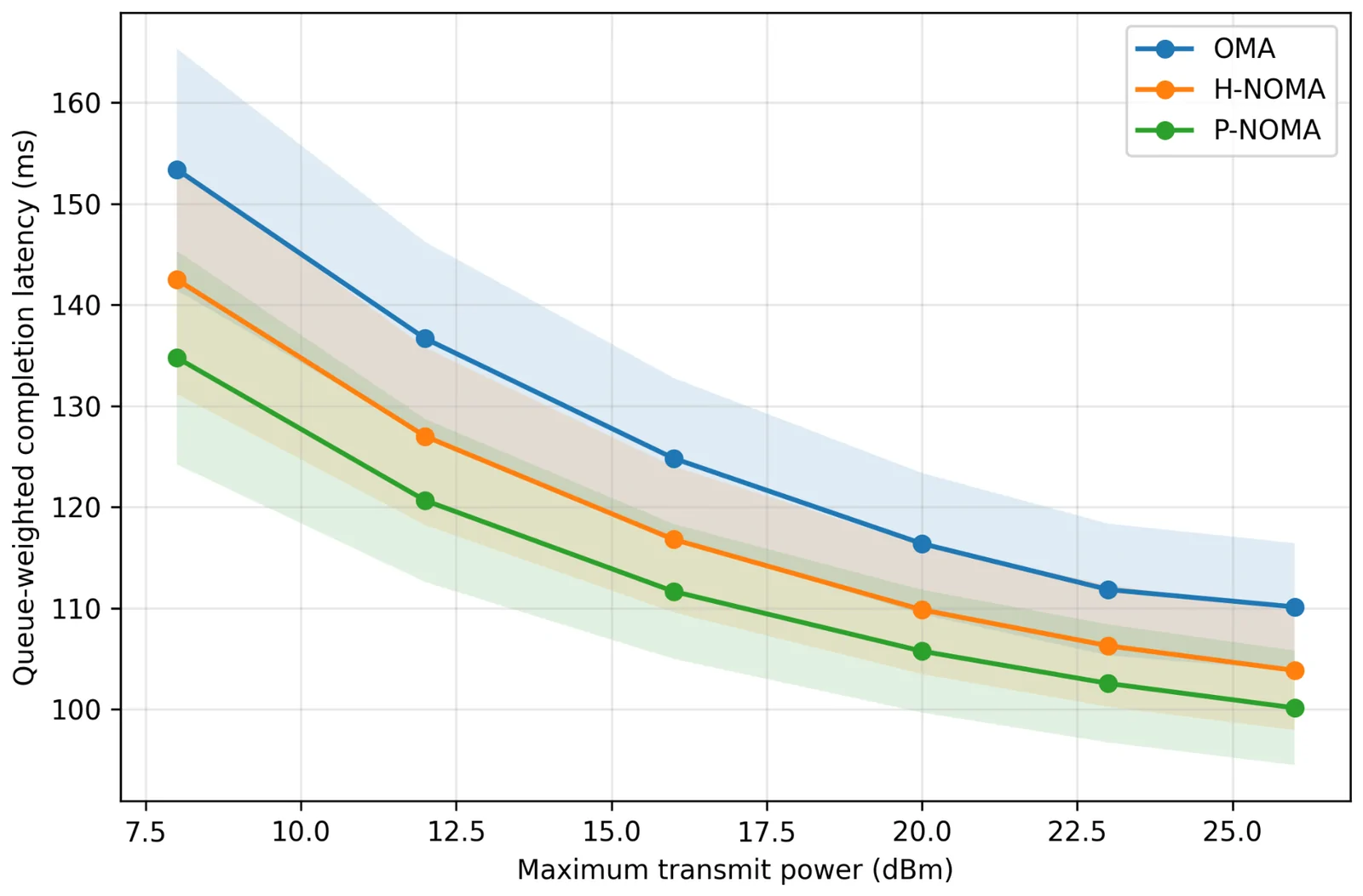
Priority-weighted task-completion latency versus maximum transmit power. Solid lines with circular markers show sample means, and shaded bands show 95% confidence intervals over 24 states per point.

**Figure 4:**
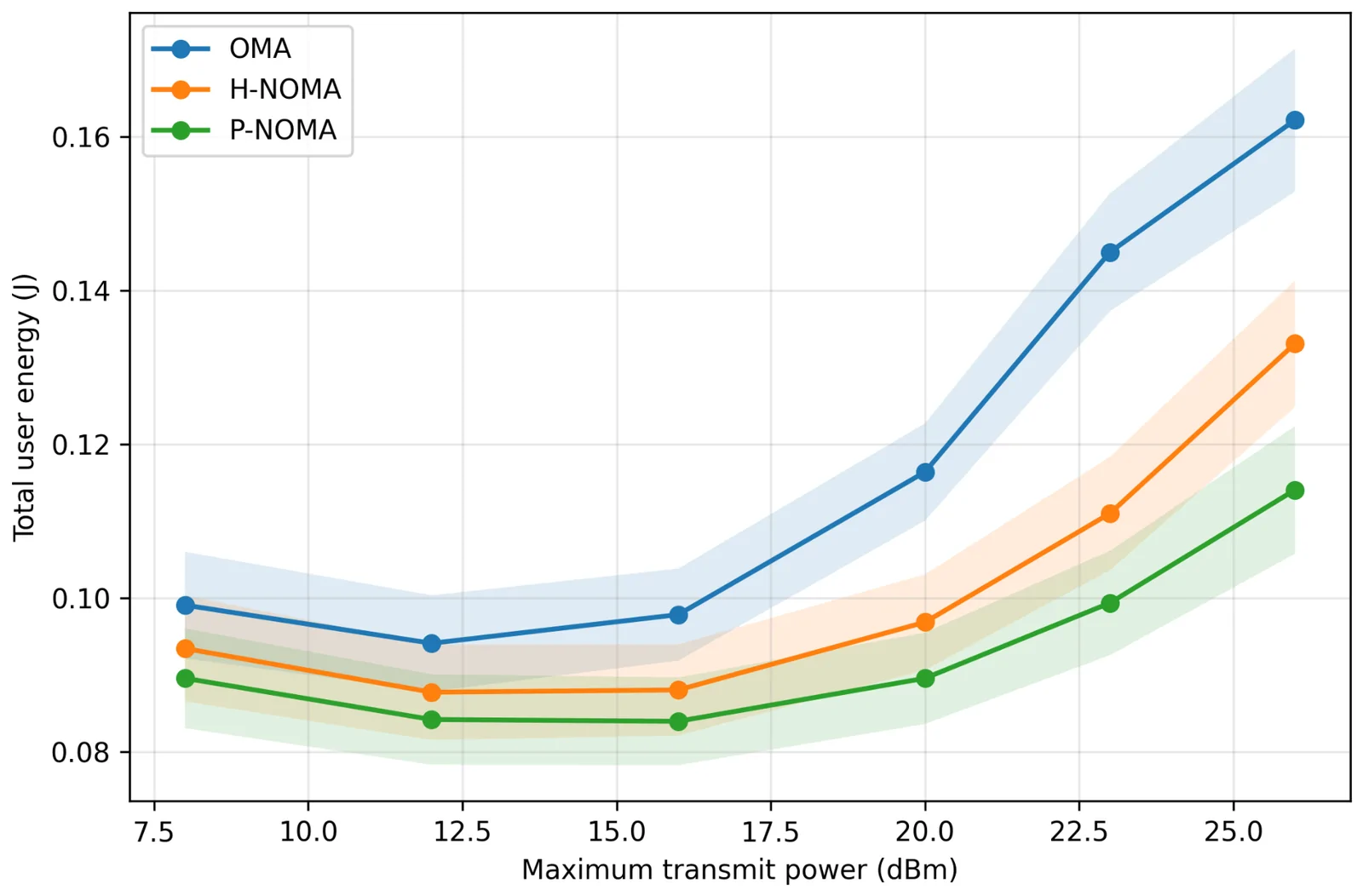
Total device energy versus maximum transmit power. Solid lines with circular markers show sample means, and shaded bands show 95% confidence intervals over 24 states per point.

Figure 2, Figure 3 and Figure 4 show the access–structure tradeoff. Increasing the power limit initially reduces transmission time and permits a more favorable task partition, whereas total device energy begins to rise beyond its minimum. P-NOMA obtains the lowest weighted cost under perfect channel information and ideal SIC because every user accesses the full 10 MHz band. H-NOMA is intermediate: each pair receives B/⌈K/2⌉ and each SIC chain contains at most two users. OMA avoids intra-group interference but allocates only B/K to each user.

Table 3 summarizes the three access modes at the default 23 dBm power limit.

At 23 dBm, H-NOMA reduces latency by 4.97%, weighted cost by 6.18%, and energy by 23.44% relative to OMA. P-NOMA yields corresponding reductions of 8.30%, 9.90%, and 31.48%. These values provide an ideal-SIC reference; cancellation errors, receiver complexity, maximum group size, and imperfect channel information may reduce the benefit of large NOMA groups.

### 7.3. Queue-Aware Service Differentiation

**Figure 5:**
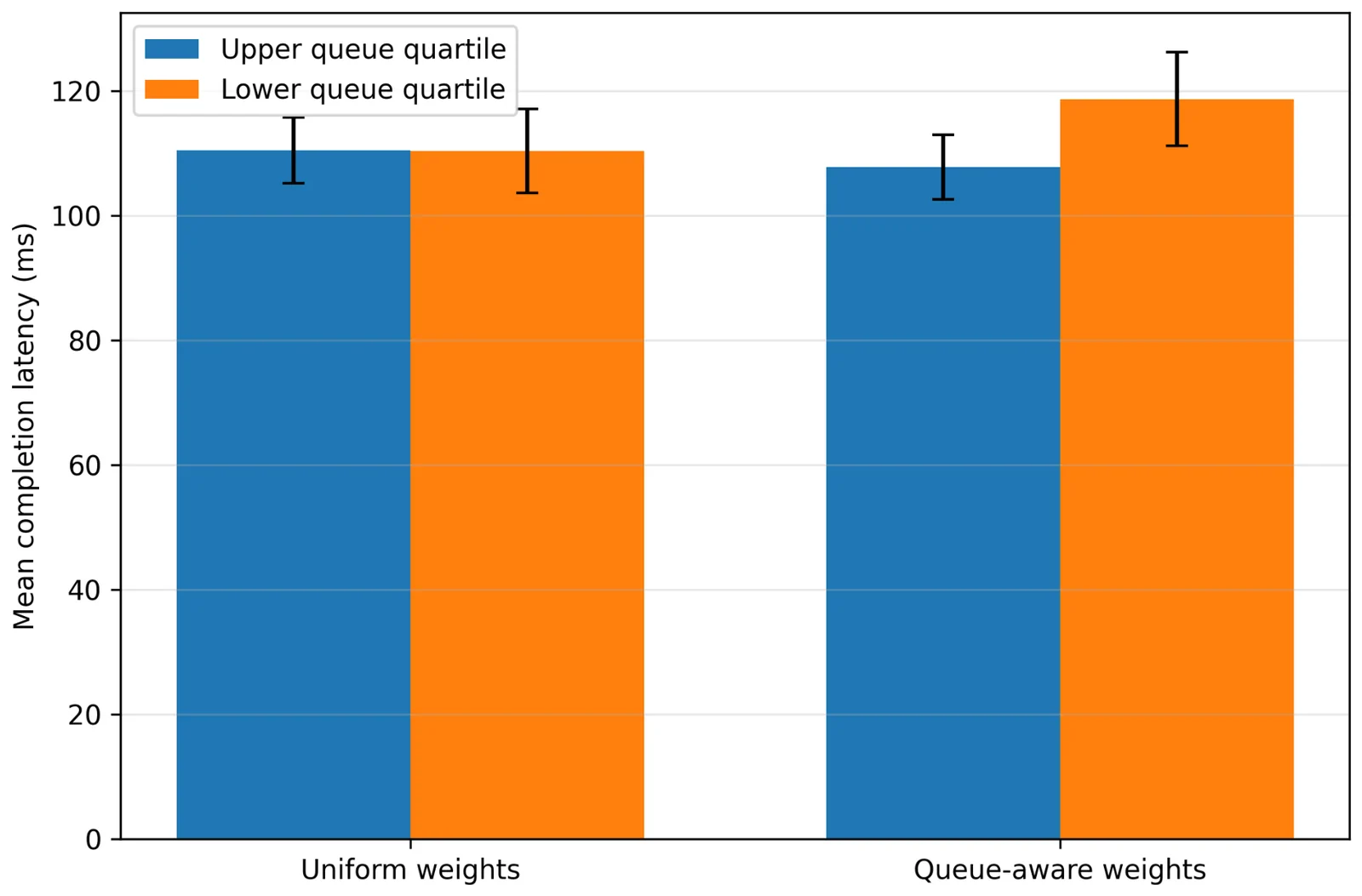
Mean completion delay for the upper and lower queue-backlog quartiles under uniform and queue-aware service weights; error bars show 95% confidence intervals over 60 states.

Figure 5 shows the effect of (2). With uniform weights, the mean delays of users in the upper and lower backlog quartiles are 110.493 and 110.390 ms, respectively. Queue-aware weighting reduces the upper-quartile mean to 107.798 ms. The paired reduction is 2.69±0.78 ms, or 2.44%. The corresponding lower-quartile mean increases to 118.731 ms; the paired penalty is 8.34±2.07 ms, or 7.56%. Thus, backlog-aware weighting provides deliberate service differentiation rather than a uniform latency improvement: backlog affects the priority assigned to a task, whereas $L_{i}$ continues to determine its transmitted and processed workload.

### 7.4. Effect of the SIC Decoding Order

**Figure 6:**
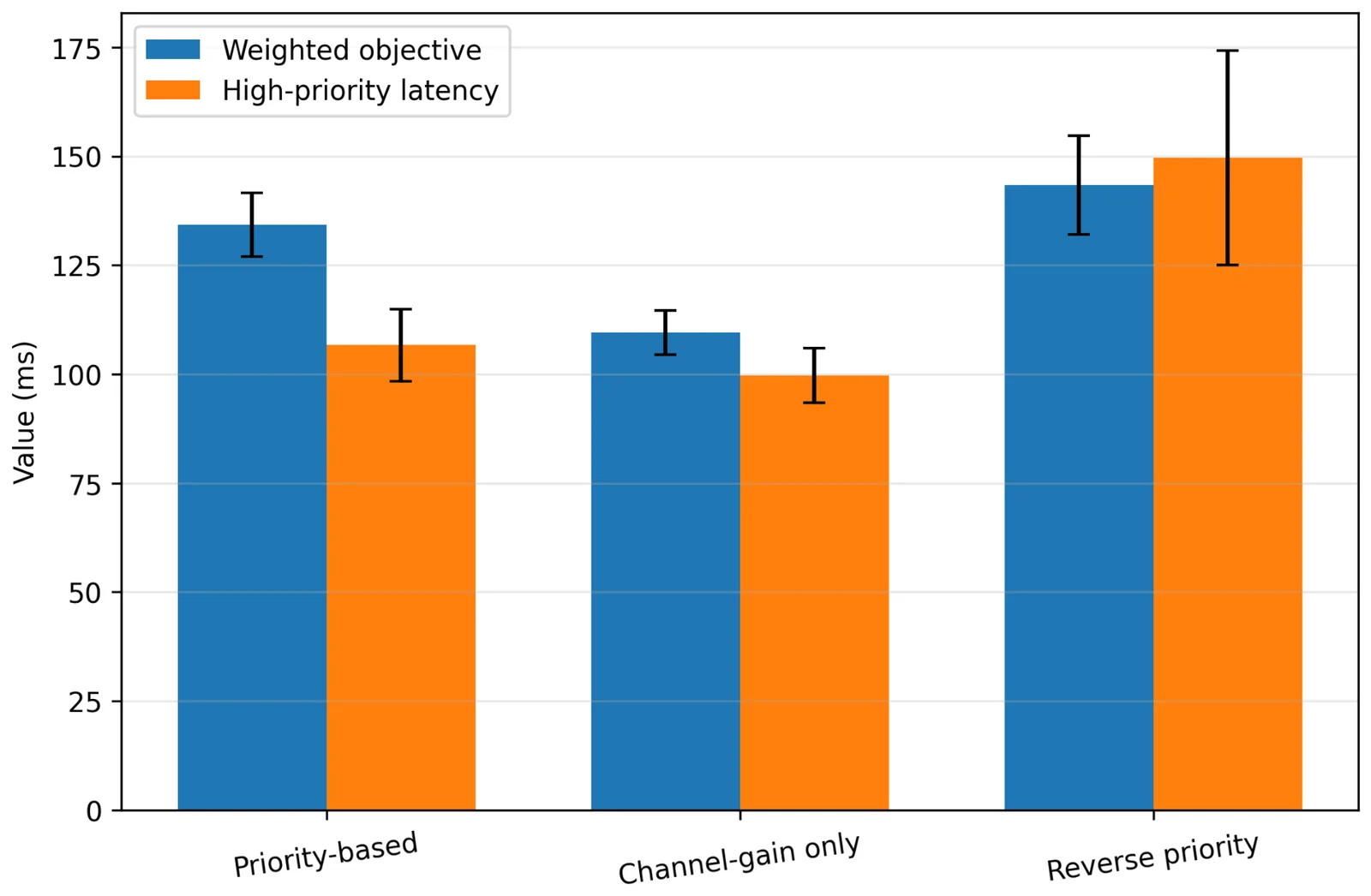
Effect of three SIC-order rules in H-NOMA on the 57 states feasible under every order; error bars show 95% confidence intervals. The priority-based rule decodes lower-weight users first, the channel-gain rule uses decreasing channel gain, and the reverse-priority rule decodes higher-weight users first.

Across all 60 generated states, the channel-gain, priority-based, and reverse-priority orders are feasible in 60, 59, and 58 cases, respectively. Figure 6 therefore compares only the 57 states feasible under all three orders. On this common set, the channel-gain rule gives a weighted cost of 109.518 ms, compared with 134.317 ms for the priority-based rule and 143.399 ms for the reverse-priority rule. The corresponding high-priority delays are 99.709, 106.669, and 149.618 ms. For the adopted strongest–weakest pairing, the channel-gain rule benefits sufficiently from interference cancellation to outperform direct priority ordering. Queue priority is therefore applied through the service weights, while the SIC order is selected from the channel conditions.

### 7.5. Convergence and Termination

**Figure 7:**
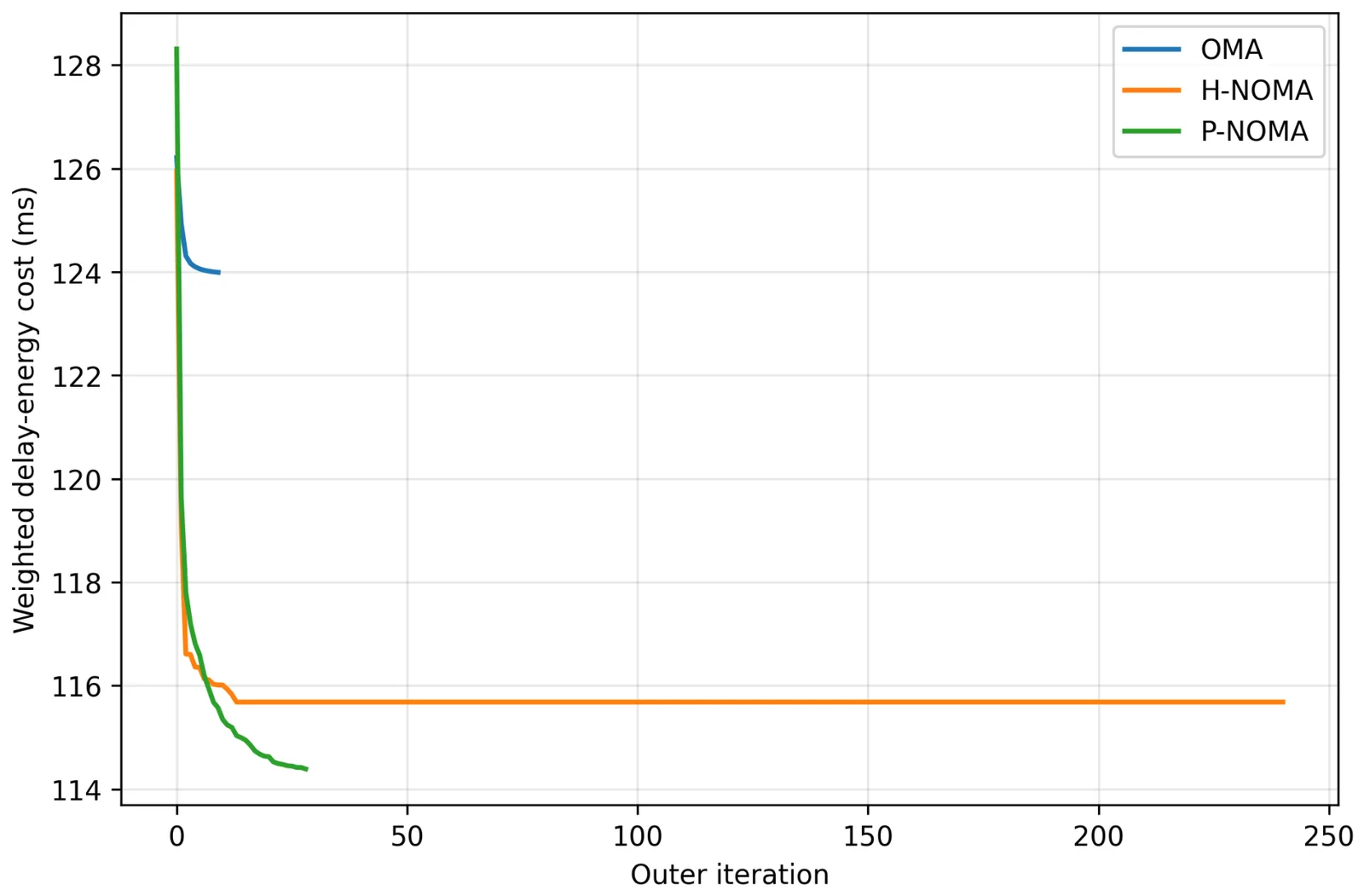
Convergence of the weighted delay–energy cost for representative SBCD-PG cases.

The representative OMA, H-NOMA, and P-NOMA cases in Figure 7 reduce the weighted cost by 1.76%, 8.14%, and 10.85%, respectively, with monotone accepted objective sequences. OMA and P-NOMA satisfy the joint window–projected-gradient test after 9 and 28 iterations. The H-NOMA case reaches the 240-iteration safeguard with $r_{\mathrm{PG}}=2.083\times 10^{-3}$, slightly above the $2\times 10^{-3}$ tolerance, and is therefore not labeled residual-converged.

Across all 432 power-sweep allocations, the mean and median iteration counts are 38.93 and 13. The joint window–projected-gradient test terminates 268 cases, the strict residual test terminates 124, and 40 reach the iteration safeguard. All 432 returned allocations are feasible. The median residual is $5.05\times 10^{-4}$, the 95th percentile is $3.16\times 10^{-3}$, and the maximum is $1.388\times 10^{-2}$. The 40 safeguard cases account for all residuals above the specified tolerance; they are retained in the performance statistics as feasible allocations but are not presented as stationary solutions.

### 7.6. Ablation Against Generic Projected Block Updates

**Figure 8:**
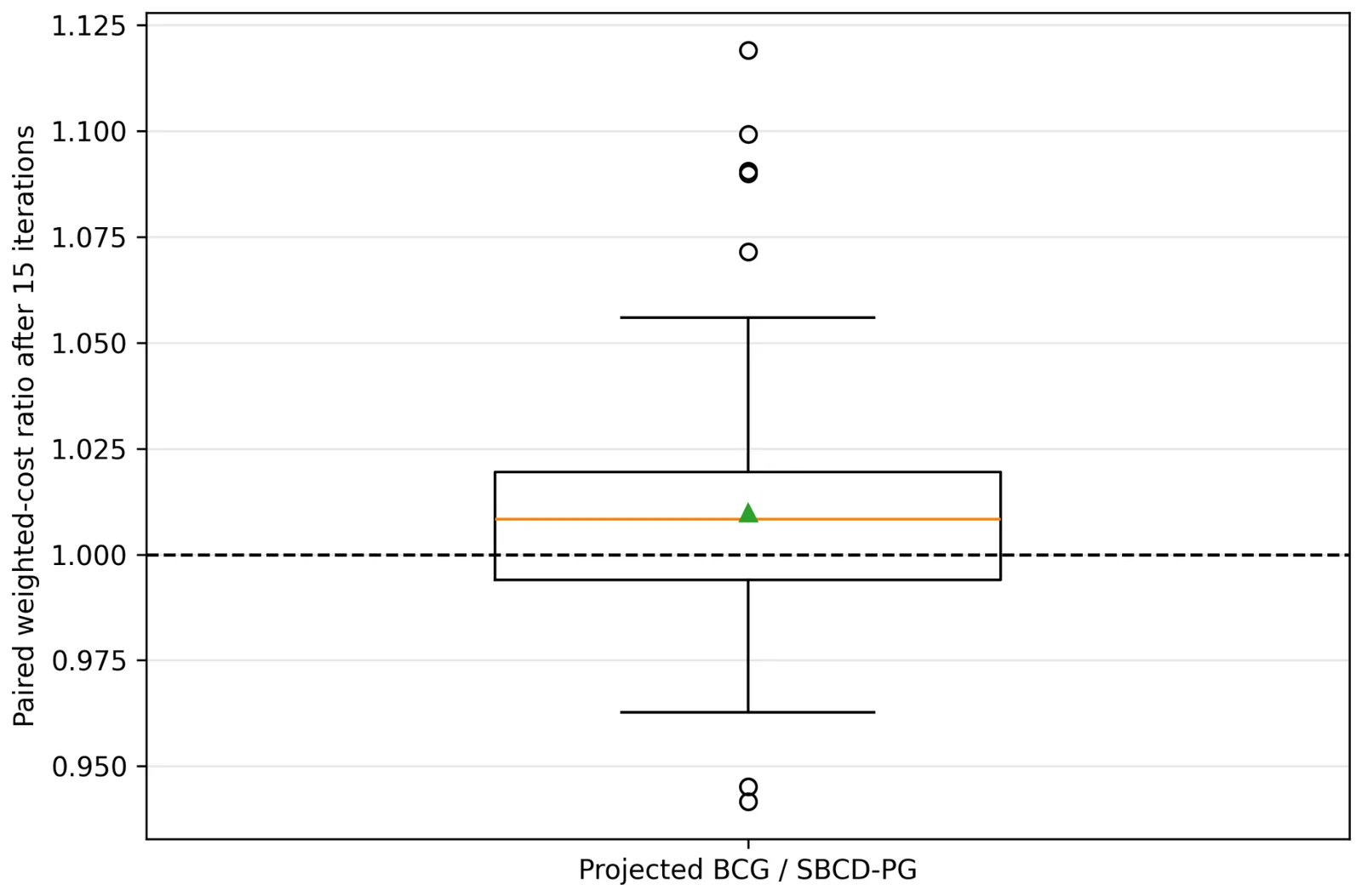
Paired weighted-cost ratio for generic projected block-coordinate updates relative to SBCD-PG after the same 15 outer iterations. The green triangle denotes the mean, the orange line denotes the median, the box spans the interquartile range, the whiskers extend to 1.5 times that range, open circles denote outliers, and the dashed horizontal line denotes equal cost.

To isolate the contribution of the exact ratio-coordinate and CPU updates, a generic projected block-coordinate gradient (PBCG) benchmark replaces each exact minimizer with one projected subgradient step. It uses the same initial raw allocation and feasibility requirements, analytical power derivative, Armijo acceptance parameters, and 15-iteration outer budget. Over 60 paired states, SBCD-PG and PBCG obtain mean weighted costs of 115.256 and 116.241 ms, respectively. The paired PBCG-to-SBCD-PG cost ratio is 1.0098±0.0086, and SBCD-PG has the lower cost in 40 of 60 states. Figure 8 therefore supports a modest improvement in objective reduction per outer iteration from exploiting the two exact updates. It does not establish universal solver or wall-clock superiority because a structured iteration performs more analytical subproblem work than a generic projected iteration.

### 7.7. Model-Based, Hybrid DDPG, and Equal Allocation

**Figure 9:**
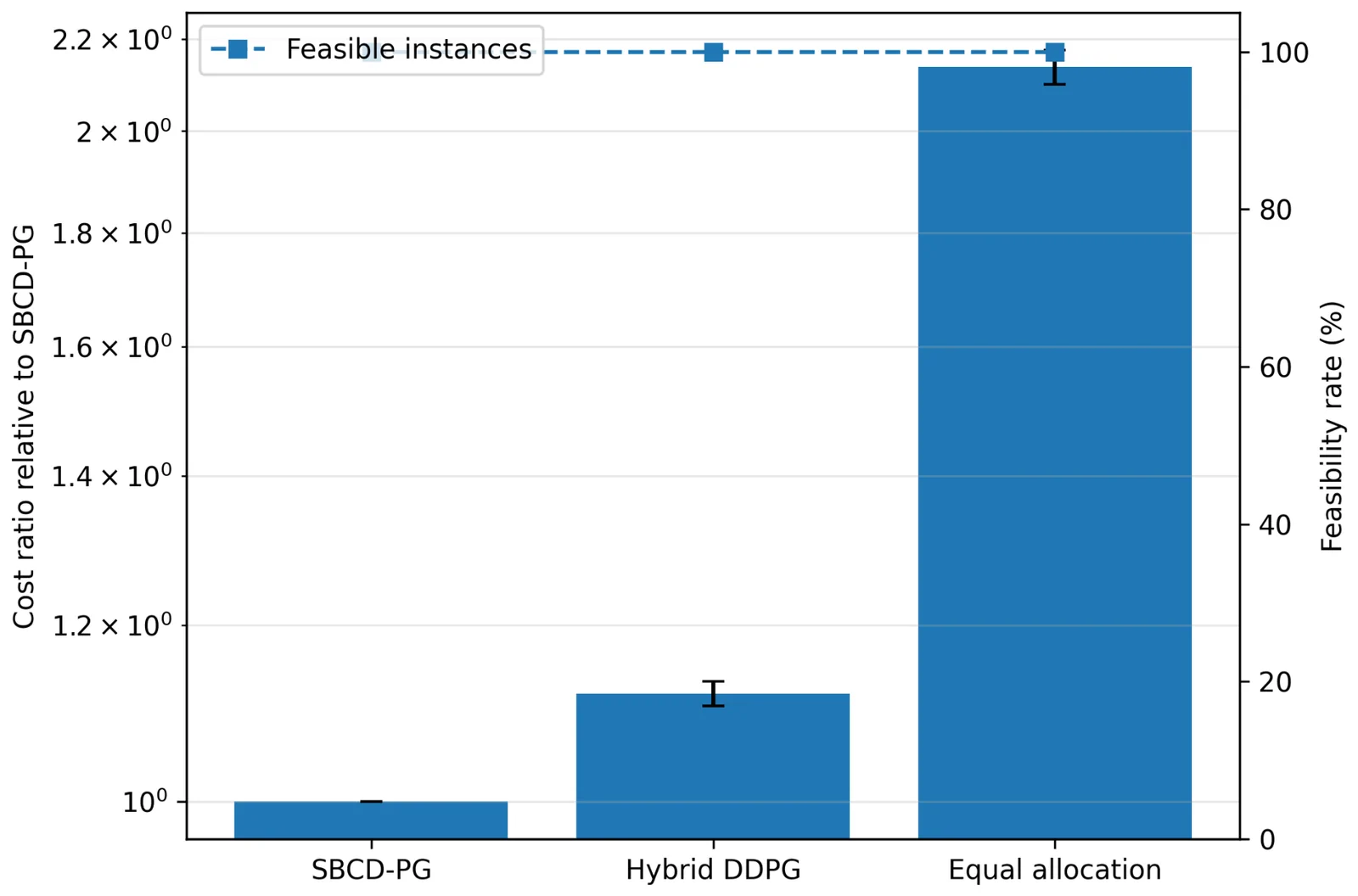
Weighted-cost ratio relative to SBCD-PG and feasibility rate over 60 test states.

Figure 9 displays the cost ratios and feasibility rates, while Table 4 reports the solution-quality and decision–time tradeoff. Averaged over the five independently trained policies, hybrid DDPG has an 11.77% higher weighted cost than SBCD-PG and requires 0.84% of its median online decision time. The mean cost ratios of the individual seeds range from 1.1088 to 1.1328, with a cross-seed standard deviation of 0.0095. Equal allocation is faster but has more than twice the SBCD-PG cost. Every method is feasible in all 60 test states; for hybrid DDPG, this statement holds for every seed–state evaluation.

**Figure 10:**
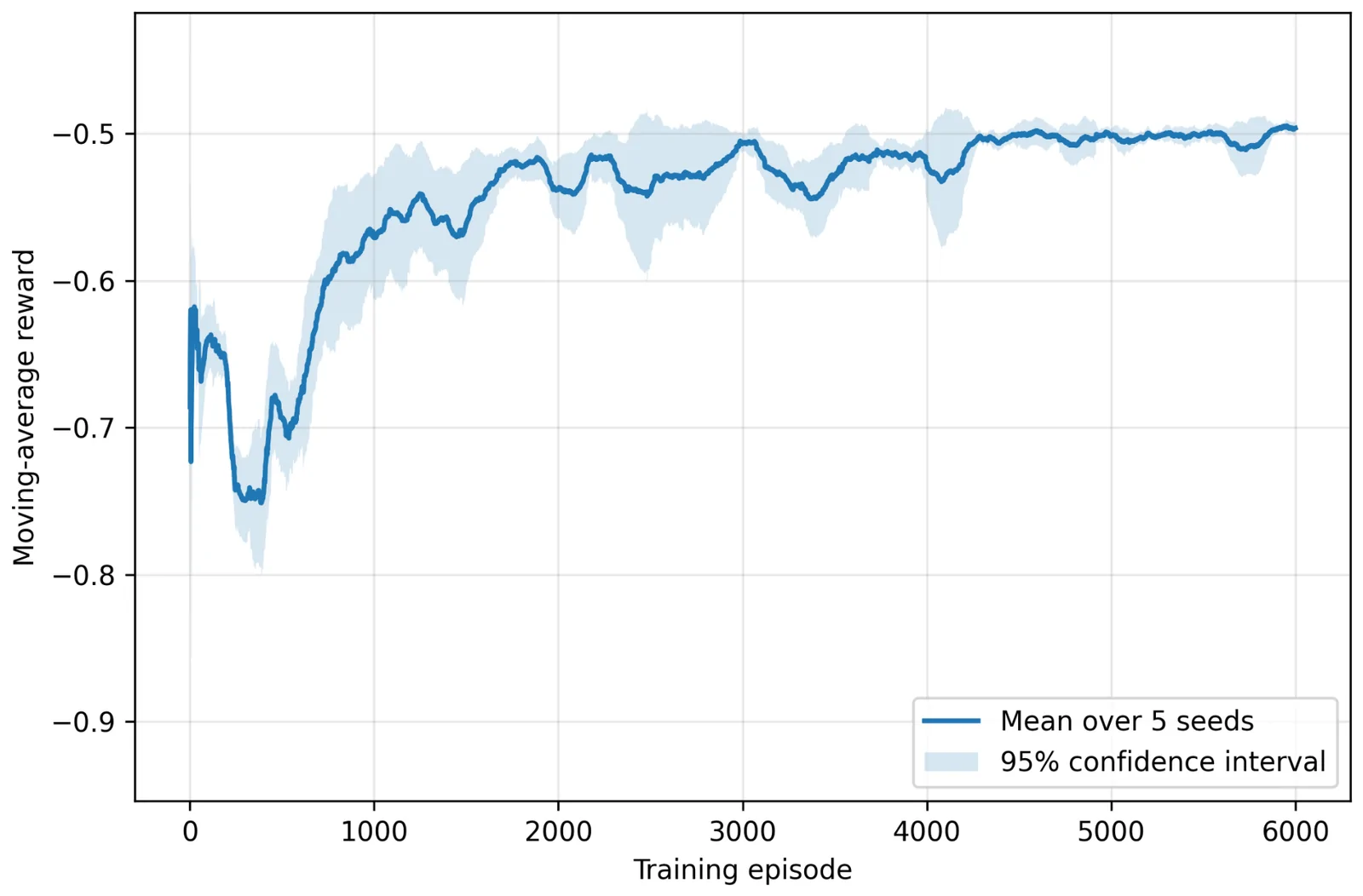
Mean moving-average terminal reward and 95% confidence band over five independent hybrid-DDPG training seeds, with 6000 episodes per seed.

The moving-average reward in Figure 10 improves over the first part of training and remains near its final level thereafter. Across the five seeds, the mean reward over the last 500 episodes is −0.5023, with a cross-seed standard deviation of 0.0099.

### 7.8. Energy–Computation Sensitivity

**Figure 11:**
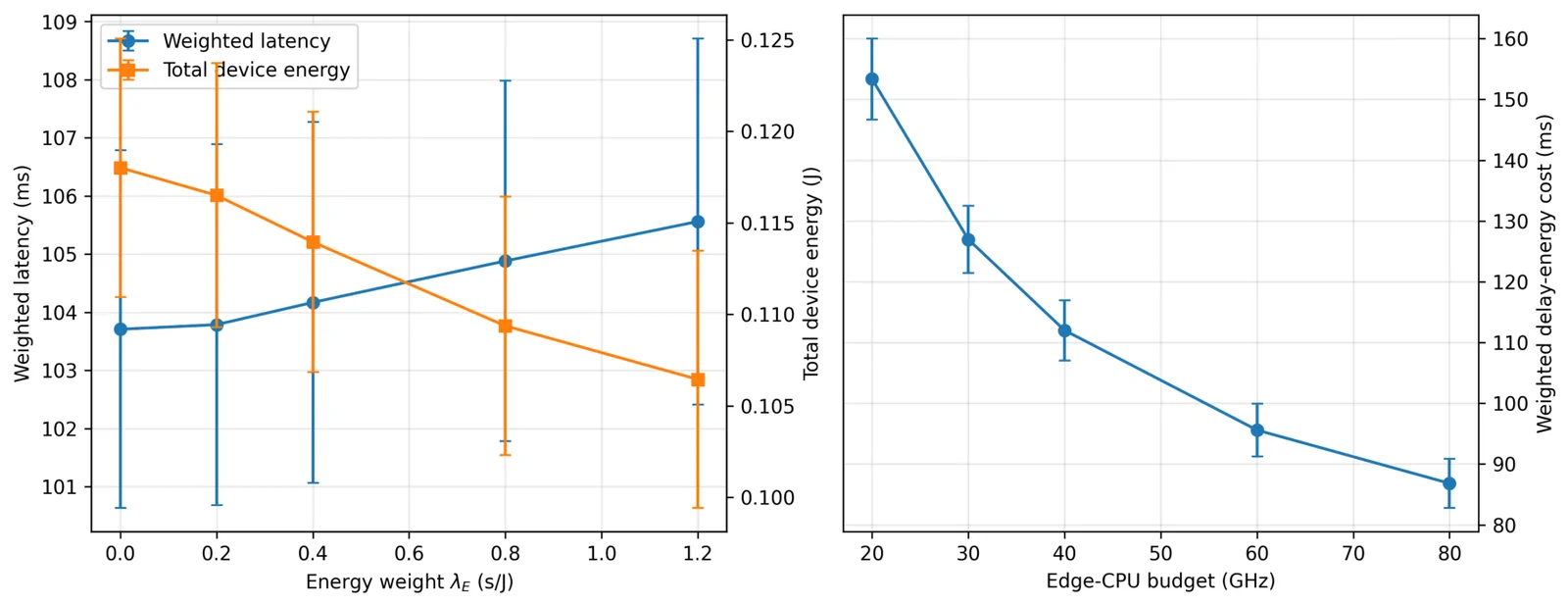
Sensitivity to the energy weight and edge-CPU budget. Points show means and 95% confidence intervals over 30 states per setting.

Figure 11 separates the objective tradeoff from the effect of edge capacity. Increasing $\lambda_{E}$ from 0 to 1.2 s/J reduces mean device energy from 0.1180 to 0.1064 J (9.80%) while increasing weighted latency from 103.709 to 105.562 ms (1.79%). Increasing $F^{e}$ from 20 to 80 GHz reduces the weighted delay–energy cost from 153.355 to 86.825 ms (43.38%). All allocations are feasible at every tested setting. These trends are consistent with the model: a larger energy weight favors lower-power operation, whereas additional edge capacity directly reduces the remote-processing component.

### 7.9. Scalability with the Number of Users

**Figure 12:**
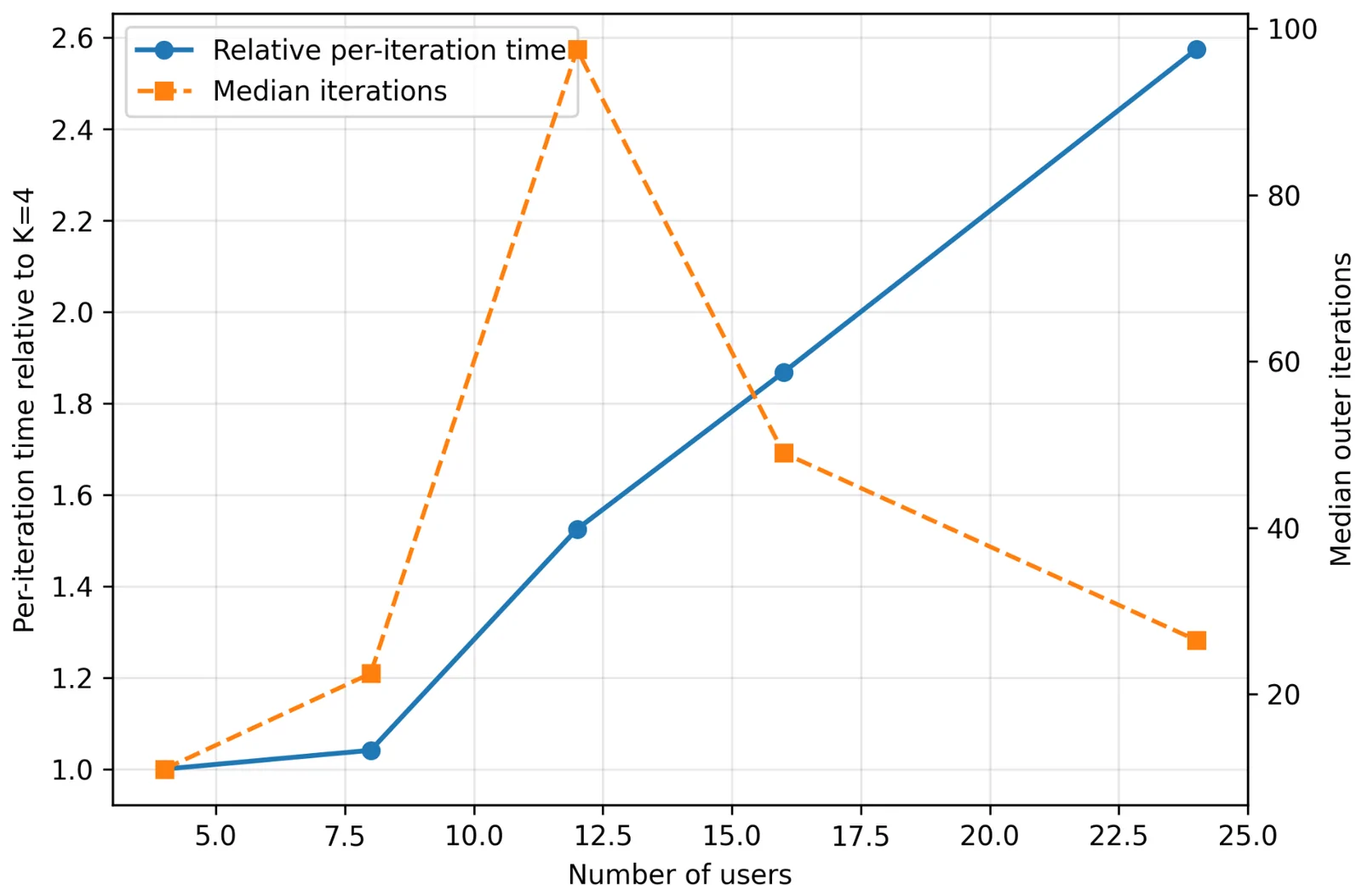
Normalized median time per outer iteration and median iteration count versus the number of users. Bandwidth and edge-CPU capacity are scaled with *K* to preserve resource density; 12 states are used per point.

Figure 12 considers K∈{4, 8, 12, 16, 24} while scaling total bandwidth and edge-CPU capacity proportionally with *K*. Relative to K=4, the median time per outer iteration is 1.04, 1.53, 1.87, and 2.57 for K=8, 12, 16,  and 24. The median outer-iteration counts are 11, 22.5, 97.5, 49, and 26.5, respectively. The non-monotone iteration counts reflect the state-dependent stopping behavior; the per-iteration growth is monotone. Every allocation in the 60-case scalability study is feasible.

### 7.10. Scope and Limitations

The results assume perfect instantaneous channel information, ideal SIC, a single cell, fixed grouping within an interval, negligible downlink-result size, and a static per-interval state. P-NOMA therefore represents an optimistic reference. SBCD-PG is a local method and does not guarantee a global optimum; moreover, an iteration-safeguard return is not a stationarity certificate. The one-step learned policy does not describe queue transitions, battery evolution, delayed channel information, or switching costs. Extensions may incorporate imperfect cancellation, robust channel uncertainty, dynamic group assignment, multiple edge servers, admission control, and time-coupled queue and battery states.

## 8. Conclusions

This paper developed a priority-aware partial-offloading framework for group-based uplink NOMA–MEC systems. Task-input size determines the transmitted and computed workload, while queue backlog and application urgency determine the service weight. The task-completion time accounts for local execution and the remote path formed by uplink transmission and edge processing.

The Gaussian-MAC rate region is convex, whereas the complete finite-CPU partial-offloading problem is not jointly convex in the adopted variables because the variable workload is coupled with reciprocal rate and reciprocal edge-CPU allocation. SBCD-PG uses exact cyclic offloading-coordinate updates, a deadline-bounded square-root CPU allocation, and an analytical projected power step. The stopping rule requires a projected-gradient condition alongside objective stagnation, and safeguard returns are identified separately from residual-qualified terminations.

For the evaluated system, pairwise H-NOMA improves the delay–energy tradeoff relative to OMA, while ideal single-group NOMA provides a lower-cost reference. Queue-aware weighting reduces the mean delay of high-backlog users at a quantified lower-backlog cost, and channel-gain SIC ordering outperforms the two priority-based alternatives on their common feasible states. The exact blocks provide a modest per-iteration advantage over generic projected block updates under a matched outer-iteration budget. The five-seed hybrid DDPG policy reduces online decision time substantially, with a moderate increase in weighted cost. Sensitivity and scalability results further show the energy–latency tradeoff, the benefit of edge capacity, and controlled per-iteration growth up to 24 users.

## Figures and Tables

**Table 1:** Positioning relative to the representative literature.

Work	MEC and Access Setting	Solution Approach	Relevance to the Present Study
[12,13]	Offloading with time and power allocation in uplink NOMA	Analytical and iterative optimization	Establishes communication–computation resource coupling
[14,15]	Partial offloading with completion-time and energy objectives in uplink NOMA	Convex special case or quasi-convex transformation	Shows that curvature depends on the task and resource model
[16,23,26]	Dynamic or secure task offloading with state-dependent allocation	DRL, DDPG, or multi-DNN	Provides learning-based allocation for continuous system states
[17,18,19]	Subchannels, hybrid OMA/NOMA access, and fairness	Problem-specific optimization	Motivates structured access and resource allocation
[20,21,22,24]	Relay, cache, wireless power, and time–energy NOMA–MEC settings	Iterative optimization and DRL	Extends NOMA–MEC to recent service and network settings
This paper	Partial offloading with finite edge CPU, queue-aware service, three access structures, and three SIC orders	Exact ratio-coordinate and deadline-bounded CPU updates, projected power update, projected-block baseline, and hybrid DDPG benchmark	Connects task demand, priority, MAC convexity, and residual-qualified allocation

**Table 2:** Default simulation and algorithm parameters.

Parameter	Value
Number of users; total bandwidth	K=8; B=10 MHz
Distance; small-scale fading	Uniform [50, 250] m; unit-mean exponential power
Noise density; receiver noise figure	−174 dBm/Hz; 7 dB
Task-input size; queue backlog	Uniform [0.40, 1.20] Mbit; uniform [0, 2.0] Mbit
Urgency factor	{1, 1.5, 2} with probabilities {0.5, 0.3, 0.2}
Local CPU; edge CPU	Uniform [0.60, 1.20] GHz; 40 GHz
Cycles per bit; deadline	500 cycles/bit; 0.80 s
Local energy coefficient; energy weight	$\kappa_{i}=10^{-28}$; $\lambda_{E}=0.40$ s/J
Queue parameters	$Q_{0}=1$ Mbit; $\eta_{Q}=3$
Power sweep	{8, 12, 16, 20, 23, 26} dBm
SBCD-PG tolerances	$\epsilon_{J}=10^{-5}$, $\epsilon_{x}=2\times 10^{-4}$, $\epsilon_{\mathrm{PG}}=2\times 10^{-3}$
Joint window–PG test; safeguard	W=5, $\epsilon_{W}=10^{-3}$; $I_{\max}=240$
Power line search; CPU bisection	$x_{\min}=10^{-3}$; initial step 0.35, shrinkage 0.5, $c_{A}=10^{-4}$; 35 bisection steps
Policy training	Five seeds; 6000 episodes/seed; batch 128; actor/critic learning rates $2\times 10^{-4}/8\times 10^{-4}$
Reward parameters	$J_{0}=0.25$ s; ζ=10

**Table 3:** Performance at $P_{i}^{\max}=23$ dBm.

Access Mode	Weighted Latency (ms)	Weighted Cost (ms)	Total Energy (J)
OMA	111.831	118.991	0.14500
H-NOMA	106.274	111.642	0.11101
P-NOMA	102.548	107.210	0.09936

**Table 4:** Allocation-method comparison for K=8 H-NOMA test states.

Method	Weighted Cost (ms)	Cost Ratio	Feasible Cases	Relative Decision Time
SBCD-PG	113.745±3.418	1.000	100%	1.0000
Hybrid DDPG	127.131±4.155	1.1177±0.0143	100%	0.0084
Equal allocation	242.595±7.507	2.1368±0.0376	100%	0.0011

## Data Availability

The data supporting the findings of this study are available from the corresponding author upon reasonable request.

## Appendix A. Fixed-SIC Individual-Rate Curvature

For a two-user group in which user 1 is decoded first,

(A1) $$R_{1}\left(p_{1},\;p_{2}\right)=\frac{B_{g}}{\ln2}\left[\ln\left(n_{g}+g_{1}p_{1}+g_{2}p_{2}\right)-\ln\left(n_{g}+g_{2}p_{2}\right)\right].$$

Let $S=n_{g}+g_{1}p_{1}+g_{2}p_{2}$ and $I=n_{g}+g_{2}p_{2}$. Its Hessian is

(A2) $$\nabla^{2}R_{1}=\frac{B_{g}}{\ln2}\left[\begin{array}{cc} -g_{1}^{2}/S^{2} & -g_{1}g_{2}/S^{2}\\ -g_{1}g_{2}/S^{2} & g_{2}^{2}\left(I^{-2}-S^{-2}\right) \end{array}\right],\;$$

with determinant

(A3) $$\det\left(\nabla^{2}R_{1}\right)=-{\left(\frac{B_{g}}{\ln2}\right)}^{2}\frac{g_{1}^{2}g_{2}^{2}}{S^{2}I^{2}}<0.$$

Thus, the individual fixed-corner rate is neither jointly concave nor jointly convex in $\left(p_{1},\;p_{2}\right)$. By contrast, the sum rate

(A4) $$R_{1}+R_{2}=B_{g}\log_{2}\left(1+\frac{g_{1}p_{1}+g_{2}p_{2}}{n_{g}}\right)$$

is jointly concave. The fixed-order derivative coupling is therefore consistent with the convexity of the complete MAC rate region.

## Appendix B. Deadline-Bounded Square-Root CPU Derivation

Ignoring terms independent of $f^{e}$, the active remote-path problem is

(A5) $$\min_{f^{e}}\;\sum_{i}\frac{w_{i}d_{i}}{f_{i}^{e}}\;s.t.\;f_{i}^{\min}\leq f_{i}^{e}\leq {\bar{f}}_{i},\;\;\sum_{i}f_{i}^{e}\leq F^{e}.$$

Let $\alpha_{i}\geq 0$ and $\beta_{i}\geq 0$ be the multipliers of the lower and upper bounds, respectively, and let ν≥0 be the budget multiplier. The stationarity condition is

(A6) $$-\frac{w_{i}d_{i}}{{\left(f_{i}^{e}\right)}^{2}}+\nu -\alpha_{i}+\beta_{i}=0,\;$$

with complementary-slackness conditions

(A7) $$\alpha_{i}\left(f_{i}^{\min}-f_{i}^{e}\right)=0,\;\;\beta_{i}\left(f_{i}^{e}-{\bar{f}}_{i}\right)=0,\;\;\nu \left(\sum_{i}f_{i}^{e}-F^{e}\right)=0.$$

For an interior user, $f_{i}^{e}=\sqrt{w_{i}d_{i}/\nu }$. Projection onto the KKT-active interval gives

(A8) $$f_{i}^{e}\left(\nu \right)=\min\left\{{\bar{f}}_{i},\;\;\max\left[f_{i}^{\min},\;\sqrt{\frac{w_{i}d_{i}}{\nu }}\right]\right\},\;$$

which is (38). The mapping $\sum_{i}f_{i}^{e}\left(\nu \right)$ is non-increasing, so scalar bisection finds the active-budget dual variable. If all active caps are finite and their sum does not exhaust the budget, the cap allocation is optimal.
